# Correlation analysis of serum amyloid A, neutrophil-lymphocyte ratio, platelet-lymphocyte ratio, and systemic immune-inflammation index with neoadjuvant therapy efficacy and prognosis in breast cancer

**DOI:** 10.3389/fonc.2026.1695123

**Published:** 2026-03-04

**Authors:** Yuanyuan Nong, Zhenzhen Zhou, Siyu Deng, Mengyu Liu, Xuefang Liang, Yonghua Jiang, Xinqing Ye, Aihua Tan

**Affiliations:** 1 Department of Breast, Bone & Soft Tissue Oncology, Guangxi Medical University Cancer Hospital, Nanning, Guangxi, China; 2 Center for Genomic and Personalized Medicine, Guangxi Key Laboratory for Genomic and Personalized Medicine, Guangxi Collaborative Innovation Center for Genomic and Personalized Medicine, Guangxi Medical University, Nanning, Guangxi, China; 3 Pathology Department, Guangxi Medical University Cancer Hospital, Nanning, Guangxi, China; 4 School of Public Health, Guangxi Medical University, Nanning, Guangxi, China; 5 Laboratory of Breast Cancer Diagnosis and Treatment Research of Guangxi Department of Education, Nanning, Guangxi, China

**Keywords:** serum amyloid A, inflammatory biomarkers, breast cancer, neoadjuvant therapy, prognosis

## Abstract

**Background:**

Previous studies have extensively explored the relationships between the neutrophil-to-lymphocyte ratio (NLR), platelet-to-lymphocyte ratio (PLR), and systemic immune-inflammation index (SII) with treatment efficacy and prognosis in breast cancer, though the conclusions have been inconsistent. Currently, research on serum amyloid A (SAA) in this context remains limited. This study aims to comprehensively evaluate the association of SAA, NLR, PLR, and SII with treatment response and prognosis in breast cancer, in order to explore which inflammatory marker may have the greatest prognosis value.

**Patients and methods:**

We retrospectively analyzed 348 breast cancer patients treated between 2019 and 2021, including 113 patients who received neoadjuvant chemotherapy. Patients were stratified based on levels of inflammatory markers (SAA: 2.06 mg/L; NLR: 2.50; PLR: 162.89; SII: 650.66). The outcomes assessed included pathological complete response (pCR) and objective response rate (ORR) to neoadjuvant therapy, event-free survival (EFS), and overall survival (OS). Statistical analyses were conducted using Log-rank tests, Cox regression, and Logistic regression.

**Results:**

Multivariate analysis identified high SAA as an independent correlate of reduced ORR (OR = 0.26, 95%CI: 0.08-0.80, p = 0.021). No inflammatory markers were found to have statistically significant correlates for pCR. For long-term prognosis, both elevated SAA and SII were independently associated with shorter OS (SAA: HR = 2.67, 95%CI: 1.14-6.26, p = 0.024; SII: HR = 2.65, 95%CI: 1.11-6.32, p = 0.028). Subgroup analysis revealed that among HER2+ patients, high SAA was independently correlated with both worse EFS (HR = 2.53, 95%CI: 1.06-6.07, p = 0.037) and OS (HR = 4.68, 95%CI: 1.11-19.70, p = 0.035). While, the independent associations of NLR and PLR with clinical outcomes were lost after adjusting for clinical confounders.

**Conclusion:**

SAA appears to be independently associated with both ORR to neoadjuvant therapy and long-term survival outcomes in breast cancer patients, particularly those with HER2+ status, when compared to NLR, PLR, and SII.

## Introduction

Breast cancer is the most prevalent malignancy among women worldwide. According to data from the International Agency for Research on Cancer (IARC), approximately 2.3 million new cases (11.6% of all cancer cases) and 666,000 deaths (6.9% of all cancer-related deaths) were reported globally in 2022 (1). Despite ongoing advancements in treatment, significant heterogeneity in patient prognosis remains. Traditional indicators, such as TNM staging and histological grade, fail to fully capture the biological complexity of tumors (2). This underscores the urgent need for novel, easily detectable, and biologically relevant biomarkers to optimize risk stratification and guide individualized treatment strategies.

Researchs over the past decade have increasingly highlighted the pivotal role of systemic inflammation in tumorigenesis, progression, and treatment resistance. Chronic inflammation drives the formation of a pro-tumor microenvironment by inducing angiogenesis, mediating immune escape, and promoting metastatic spread (3–5). In breast cancer, a “vicious cycle” exists between the local tumor and systemic inflammation: inflammation drives tumor progression, which in turn exacerbates the systemic inflammatory response. This bidirectional interplay positions systemic inflammatory biomarkers as promising tools for prognosis prediction, capable of integrating information from both the local tumor microenvironment and the systemic immune status.

Numerous studies have explored the prognostic value of inflammatory markers in breast cancer, including the neutrophil-to-lymphocyte ratio (NLR), platelet-to-lymphocyte ratio (PLR), and systemic immune-inflammation index (SII) (6–9). These markers are easily accessible through routine blood tests, making them potentially suitable for clinical application. However, their predictive value varied across studies: for example, some researchs reported NLR as a predictor of poor prognosis in locally advanced breast cancer (8), while others found no significant association with disease-free survival in patients with Luminal subtypes (7). Furthermore, these cell-count-based ratios lack specificity for particular inflammatory pathways, which may limit their utility as independent predictive indicators. Given the complex regulatory network between immunity, inflammation, and tumor biology, a biomarker capable of comprehensively reflecting the systemic inflammatory state might offer better stability and predictive power.

Serum amyloid A (SAA) is an acute-phase protein primarily synthesized by the liver in response to pro-inflammatory cytokines (e.g., IL-6, TNF-α), and it can also be aberrantly expressed in tumor cells and metastatic sites (10). Unlike NLR, PLR, or SII, SAA is an acute-phase protein with direct tumor-promoting functions. It not only reflects the systemic inflammatory status but also directly participates in tumor progression and immune regulation by activating signaling pathways such as TLR4/NF-κB (10–12). Prior studies have demonstrated that SAA may be a prognostic marker in various cancers, including pancreatic cancer (10), hepatocellular carcinoma (13), and non-small cell lung cancer (14). In breast cancer, cohort studies have shown that elevated SAA levels are associated with worse recurrence-free survival (15) and are linked to estrogen receptor-negative subtypes (16). However, its association with response to neoadjuvant therapy and its prognostic significance across different molecular subtypes, such as Human Epidermal Growth Factor Receptor 2 (HER2)-positive(HER2+) versus HER2-negative(HER2-) breast cancer, remain to be systematically investigated. This study aims to comprehensively evaluate the association of SAA and these traditional inflammatory markers with neoadjuvant treatment response and long-term prognosis in breast cancer.

## Method

### Patients

This study conducted a retrospective analysis of breast cancer patients treated at Guangxi Medical University Cancer Hospital between September 11, 2019, and September 24, 2021. Eligible patients had a histopathological diagnosis of breast cancer, had not received any prior anticancer treatment, and tested negative for Severe Acute Respiratory Syndrome Coronavirus 2 (SARS-CoV-2) nucleic acid upon admission. Only patients with complete clinical, pathological, and follow-up data, as well as eligible blood samples, were included. Patients with concurrent inflammatory breast cancer, autoimmune or hematological disorders, a history of other malignancies, or other acute or chronic infectious diseases were excluded. Ultimately, 348 patients met the criteria for analysis.

### Blood sample collection and testing

All blood samples were collected prior to the initiation of anticancer treatment, with serum separated and stored in a -80°C freezer after collection. SAA testing was conducted uniformly during the same time period, using the method of sandwich enzyme-linked immunosorbent assay (ELISA). The complete blood count was performed using a fully automated hematology analyzer at the hospital.

### Data collection

Clinical and pathological data were extracted from electronic medical records, including age at diagnosis, tumor histological type, primary tumor size (T stage), lymph node status (N stage), metastasis status (M stage), TNM stage (according to the 8th edition AJCC guidelines (17)), estrogen receptor (ER)/progesterone receptor (PR) expression status (positive defined as nuclear staining in ≥1% of tumor cells), human epidermal growth factor receptor 2 (HER2) status (immunohistochemistry [IHC] 3+ or IHC 2+ with positive fluorescence *in situ* hybridization [FISH] defined as HER2+ (18, 19)), treatment regimen, and laboratory parameters. Inflammatory indices were calculated as follows: NLR = absolute neutrophil count/absolute lymphocyte count; PLR = absolute platelet count/absolute lymphocyte count; SII = (neutrophil count × platelet count)/lymphocyte count (all counts in ×10^9^/L). Treatment response was evaluated per RECIST 1.1 criteria, and pathological response (pCR) was assessed using the Miller-Payne (MP) grading system. The total of partial response (PR) and complete response (CR) is known as the objective response rate (ORR). Neoadjuvant treatment was administered based on molecular subtype (see detailed regimens in **Supplementary Table 2**). HER2+ patients received anti−HER2 targeted therapy combined with chemotherapy. HER2- with hormone receptor−positive disease received chemotherapy or endocrine therapy, while triple-negative breast cancer (TNBC) patients were treated with chemotherapy. Event-free survival (EFS) was defined as the time from the initiation of treatment to tumor progression, recurrence, or death from any cause. Overall survival (OS) was defined as the time from the date of histological diagnosis to death from any cause. The study was approved by the Ethics Committee of Guangxi Medical University Cancer Hospital (Approval No: KY2022395), As the data were anonymized and the study complied with the principles of the Declaration of Helsinki, informed consent was waived.

### Follow-up

Follow-up was conducted through telephone interviews, with data recorded in the hospital’s electronic medical record system. The cutoff date for follow-up was July 5, 2024, and cases lost to follow-up were treated as censored data in the analysis.

### Statistical analysis

Continuous variables conforming to a normal distribution are presented as mean ± standard deviation (Mean ± SD). Non-normally distributed variables are presented as median (interquartile range) [M (Q_1_, Q_3_)]. Categorical variables are presented as frequency (percentage) [n (%)]. Group comparisons were performed using the chi-square test, independent samples t-test, or Mann-Whitney U test. Based on the upper tertile, patients were stratified into high and low expression groups for SAA (2.06 mg/L), NLR (2.50), PLR (162.89), and SII (650.66). Survival curves were plotted using the Kaplan-Meier method, and differences between groups were compared using the log-rank test. To address multicollinearity among the related inflammatory indices (SII, NLR, PLR), we compared several pre-specified multivariable Cox models, each containing different combinations of these dichotomized markers. The model with the best fit, determined by the lowest Akaike Information Criterion (AIC) and the highest Concordance Index (C-index), was selected as the final model (**Supplementary Tables 4**-**7**). Results from univariate and multivariate Cox regression analyses (variables with P < 0.10 or an effect size change > 10% in univariate analysis were included in the multivariate model) are presented as hazard ratios (HR) with 95% confidence intervals (CI). The efficacy of neoadjuvant therapy was analyzed using logistic regression, with results presented as odds ratios (OR) with 95% CI. All analyses were performed using SPSS version 27.0.1 and R version 4.4.3 software, and P value < 0.05 was considered statistically significant.

## Result

### Baseline characteristics

A total of 348 patients were included in the analysis (**Table 1**). The cohort consisted of 71%, 18%, and 11% with stage I-II, III, and IV disease, respectively. The high SAA group (SAA > 2.06 mg/L) had a higher proportion of ER+ cases (66% vs. 53%, p = 0.022) and a lower proportion of HER2+ cases (37% vs. 50%, p = 0.018) compared to the low SAA group. NLR, PLR, and SII were closely linked to tumor burden. Elevated NLR (NLR > 2.50) was associated with advanced T stage (29% vs. 18%, p = 0.025), N stage (22% vs. 12%, p = 0.015), and stage III disease (28% vs. 14%, p = 0.007). High PLR (PLR > 162.89) was related to increased N2/N3 nodal involvement (23% vs. 11%, p = 0.002). High SII showed a borderline link to stage III disease (25% vs. 15%, p = 0.066).

**Table 1:** Baseline characteristics of breast cancer patients.

Variable	N	SAA ≤ 2.06mg/L N = 232	SAA>2.06mg/L N = 116	p-value	NLR ≤ 2.50 N = 232	NLR>2.50 N = 116	p-value	PLR ≤ 162.89 N = 232	PLR>162.89 N = 116	p-value	SII ≤ 650.66 N = 232	SII>650.66 N = 116	p-value
Age, median(Q1,Q3), years	348	47 (40, 56)	50 (42, 57)	0.112	47 (41, 56)	49 (42, 57)	0.245	47 (40, 56)	48 (42, 55)	0.477	48 (41, 56)	48 (40, 55)	0.940
BMI, median(Q1,Q3)	348	22.20 (20.40, 24.50)	22.60(20.90, 24.80)	0.141	22.20(20.70, 24.50)	22.40 (20.40, 24.70)	0.773	22.20 (20.70, 24.50)	22.60 (20.30, 24.70)	0.885	22(20.60, 24.40)	22.80 (20.60, 25.20)	0.138
T stage, n (%)	340			0.941			0.025			0.164			0.146
≤T2		178 (78%)	89 (79%)		187 (82%)	80 (71%)		184 (81%)	83 (74%)		185 (81%)	82 (74%)	
≥T3		49 (22%)	24 (21%)		41 (18%)	32 (29%)		44 (19%)	29 (26%)		44 (19%)	29 (26%)	
N stage, n (%)	348			0.832			0.015			0.002			0.137
<N2		198 (85%)	98 (84%)		205 (88%)	91 (78%)		207 (89%)	89 (77%)		202 (87%)	94 (81%)	
≥N2		34 (15%)	18 (16%)		27 (12%)	25 (22%)		25 (11%)	27 (23%)		30 (13%)	22 (19%)	
M stage, n (%)	348			0.390			0.810			0.390			0.330
M0		209 (90%)	101 (87%)		206 (89%)	104 (90%)		209 (90%)	101 (87%)		204 (88%)	106 (91%)	
M1		23 (10%)	15 (13%)		26 (11%)	12 (10%)		23 (10%)	15 (13%)		28 (12%)	10 (9%)	
TNM, n (%)	348			0.568			0.007			0.131			0.066
I-II		168 (72%)	78 (67%)		174 (75%)	72 (62%)		172 (74%)	74 (64%)		169 (73%)	77 (66%)	
III		41 (18%)	23 (20%)		32 (14%)	32 (28%)		37 (16%)	27 (23%)		35 (15%)	29 (25%)	
IV		23 (10%)	15 (13%)		26 (11%)	12 (10%)		23 (10%)	15 (13%)		28 (12%)	10 (9%)	
Ki67, n (%)	344			0.073			0.880			0.790			0.880
<40%		106 (46%)	65 (57%)		114 (50%)	57 (49%)		115 (50%)	56 (49%)		114 (50%)	57 (49%)	
≥40%		123 (54%)	50 (43%)		114 (50%)	59 (51%)		114 (50%)	59 (51%)		114 (50%)	59 (51%)	
ER status, n (%)	348			0.022			0.251			0.169			0.491
Negative		110 (47%)	40 (34%)		105 (45%)	45 (39%)		94 (41%)	56 (48%)		103 (44%)	47 (41%)	
Positive		122 (53%)	76 (66%)		127 (55%)	71 (61%)		138 (59%)	60 (52%)		129 (56%)	69 (59%)	
PR status, n (%)	348			0.081			0.289			0.225			0.404
Negative		125 (54%)	51 (44%)		122 (53%)	54 (47%)		112 (48%)	64 (55%)		121 (52%)	55 (47%)	
Positive		107 (46%)	65 (56%)		110 (47%)	62 (53%)		120 (52%)	52 (45%)		111 (48%)	61 (53%)	
HER2 status, n (%)	348			0.018			0.224			0.287			0.148
Negative		115 (50%)	73 (63%)		120 (52%)	68 (59%)		130 (56%)	58 (50%)		119 (51%)	69 (59%)	
Positive		117 (50%)	43 (37%)		112 (48%)	48 (41%)		102 (44%)	58 (50%)		113 (49%)	47 (41%)	
Responder, n (%)	113			0.024			0.947			0.417			0.566
ORR		64 (83%)	23 (64%)		63 (77%)	24 (77%)		64 (79%)	23 (72%)		62 (78%)	25 (74%)	
Non-ORR		13 (17%)	13 (36%)		19 (23%)	7 (23%)		17 (21%)	9 (28%)		17 (22%)	9 (26%)	
Pathological response, n (%)	106			0.269			0.761			0.693			0.351
pCR		34 (46%)	11 (34%)		32 (42%)	13 (45%)		34 (44%)	11 (39%)		34 (45%)	11 (35%)	
Non-pCR		40 (54%)	21 (66%)		45 (58%)	16 (55%)		44 (56%)	17 (61%)		41 (55%)	20 (65%)	

SAA, serum amyloid A; NLR, neutrophil/lymphocyte ratio; PLR, platelet/lymphocyte ratio; SII, neutrophil ×platelet/lymphocyte ratio; BMI, Body Mass Index; TNM, Tumor-Node-Metastasis staging system; ER, Estrogen Receptor; PR, Progesterone Receptor; HER2, Human Epidermal Growth Factor Receptor 2; ORR (objective response rate), Complete response (CR) + Partial response (PR); pCR, pathological complete response.

### Association of inflammatory markers with neoadjuvant treatment response

Among the 348 patients, 113 received more than two cycles of neoadjuvant therapy and were eligible for efficacy evaluation. Of these, 106 had pathological MP grading data available for pCR analysis. Among the four inflammatory markers, only SAA showed a significant association with treatment response. High SAA was correlated with a lower ORR (64% vs. 83%, p = 0.024). Additionally, patients with high SAA tended to have a lower pCR rate, although this difference was not statistically significant (34% vs. 46%, p = 0.269). NLR, PLR, and SII showed no significant associations with either ORR or pCR (**Table 1**). In multivariate analysis, after adjusting for clinical confounders, high SAA remained independently associated with lower ORR (OR = 0.26, 95% CI: 0.08-0.80, p = 0.021) (**Table 2**).

**Table 2:** Logistic regression analysis for neoadjuvant treatment response.

Variable	ORR					PCR				
	N	Univariate analysis		Multivariate analysis		N	Univariate analysis		Multivariate analysis	
		OR (95%CI)	p-value	OR (95%CI)	p-value		OR (95%CI)	p-value	OR (95%CI)	p-value
Age, years	113	0.96 (0.92, 1.01)	0.114			106	0.95 (0.91, 1.00)	0.037	0.95 (0.90, 1.00)	0.052
BMI	113	1.02 (0.89, 1.16)	0.814			106	1.02 (0.91, 1.15)	0.718		
T stage										
≤T2	83	1				76	1			
≥T3	30	0.76 (0.30, 2.08)	0.579			30	0.72 (0.29, 1.69)	0.450		
N stage										
<N2	100	1		1		93	1			
≥N2	13	0.29 (0.09, 0.99)	0.043	0.40 (0.08, 1.95)	0.253	13	0.56 (0.14, 1.86)	0.368		
TNM										
I-II	79	1				73	1		1	
III	34	0.49 (0.20, 1.24)	0.125			33	0.47 (0.19, 1.11)	0.092	0.39 (0.13, 1.07)	0.075
Ki67										
<40%	50	1		1		44	1		1	
≥40%	61	2.98 (1.21, 7.73)	0.020	3.39 (1.14, 11.00)	0.033	60	2.14 (0.96, 4.92)	0.066	2.36 (0.93, 6.34)	0.078
ER status										
Negative	58	1				55	1			
Positive	55	0.62 (0.25, 1.50)	0.296			51	0.57 (0.26, 1.23)	0.153		
PR status										
Negative	66	1				63	1		1	
Positive	47	0.64 (0.26, 1.56)	0.323			43	0.50 (0.22, 1.10)	0.091	0.45 (0.16, 1.18)	0.111
HER2 status										
Negative	49	1		1		44	1		1	
Positive	64	12.20 (4.20, 44.90)	<0.001	13.80 (4.33, 56.40)	<0.001	62	5.38 (2.28, 13.70)	<0.001	5.68 (2.23, 16.00)	<0.001
SAA										
≤2.06mg/L	77	1		1		74	1			
>2.06mg/L	36	0.36 (0.14, 0.89)	0.027	0.26 (0.08, 0.80)	0.021	32	0.62 (0.25, 1.44)	0.270		
NLR										
≤2.50	82	1				77	1			
>2.50	31	1.03 (0.40, 2.93)	0.947			29	1.14 (0.48, 2.70)	0.762		
PLR										
≤162.89	81	1				78	1			
>162.89	32	0.68 (0.27, 1.79)	0.418			28	0.84 (0.34, 2.00)	0.693		
SII										
≤650.66	79	1				75	1			
>650.66	34	0.76 (0.30, 1.99)	0.567			31	0.66 (0.27, 1.56)	0.352		

CI, Confidence Interval; OR, Odds Ratio; SAA, serum amyloid A; NLR, neutrophil/lymphocyte ratio; PLR, platelet/lymphocyte ratio; SII, neutrophil ×platelet/lymphocyte ratio; BMI, Body Mass Index; TNM, Tumor-Node-Metastasis staging system; ER, Estrogen Receptor; PR, Progesterone Receptor; HER2, Human Epidermal Growth Factor Receptor 2; ORR (objective response rate), Complete response (CR) + Partial response (PR); pCR, pathological complete response.

Subgroup analysis by HER2 status revealed no statistically significant associations between inflammatory markers and pCR. However, HER2+ patients with low inflammatory status showed a trend toward higher pCR rates (SAA: 64% vs. 44%; SII: 63% vs. 38%), a pattern not observed in HER2- patients (**Supplementary Table 1**).

### Correlation of inflammatory markers with EFS

**Figure 1:**
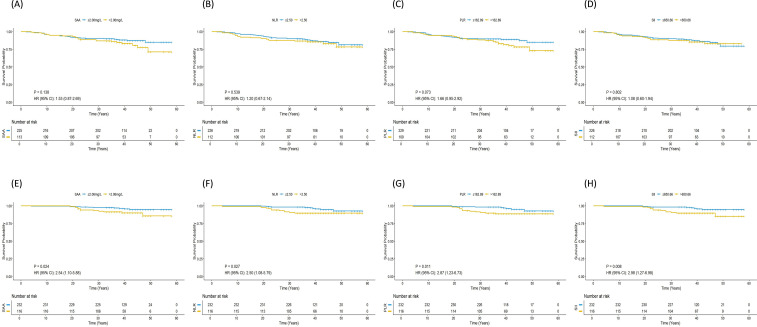
Kaplan-Meier survival analysis for event-free survival (EFS) and overall survival (OS) in breast cancer patients stratified by systemic inflammatory biomarkers. **(A–D)** EFS curves based on serum amyloid A (SAA), neutrophil-to-lymphocyte ratio (NLR), platelet-to-lymphocyte ratio (PLR), and systemic immune-inflammation index (SII) levels. **(E–H)** Corresponding OS curves for the same biomarkers.

The EFS analysis included 338 patients, with 10 excluded due to loss to follow-up. In the overall cohort, none of the inflammatory markers (SAA, NLR, PLR, SII) were significantly associated with EFS in either univariate or multivariate analysis. (**Table 3**, **Figure 1**).

**Table 3:** Univariate and multivariate analyses of EFS.

Variable	N	Univariate analysis		Multivariate analysis	
		HR (95%CI)	p-value	HR (95%CI)	p-value
Age, years	338	1.00 (0.98, 1.03)	0.811		
BMI	338	0.93 (0.85, 1.02)	0.108		
T stage					
≤T2	261	1		1	
≥T3	69	3.32 (1.89, 5.84)	<0.001	1.52 (0.77, 2.99)	0.224
N stage					
<N2	290	1		1	
≥N2	48	4.60 (2.59, 8.17)	<0.001	1.84 (0.88, 3.85)	0.103
M stage					
M0	304	1		1	
M1	34	7.33 (4.12, 13.00)	<0.001	4.09 (1.93, 8.69)	<0.001
Ki67					
<40%	166	1			
≥40%	168	0.98 (0.56, 1.72)	0.950		
ER status					
Negative	143	1			
Positive	195	0.85 (0.48, 1.50)	0.572		
PR status					
Negative	169	1			
Positive	169	0.77 (0.43, 1.37)	0.375		
HER2 status					
Negative	183	1			
Positive	155	0.93 (0.53, 1.63)	0.790		
SAA					
≤2.06mg/L	225	1			
>2.06mg/L	113	1.53 (0.87, 2.69)	0.141		
NLR					
≤2.50	226	1			
>2.50	112	1.20 (0.67, 2.14)	0.539		
PLR					
≤162.89	229	1		1	
>162.89	109	1.66 (0.95, 2.92)	0.077	1.41 (0.78, 2.54)	0.255
SII					
≤650.66	226	1			
>650.66	112	1.08 (0.60, 1.94)	0.800		

CI, Confidence Interval; HR, Hazard Ratio; SAA, serum amyloid A; NLR, neutrophil/lymphocyte ratio; PLR, platelet/lymphocyte ratio; SII, neutrophil ×platelet/lymphocyte ratio; BMI, Body Mass Index; ER, Estrogen Receptor; PR, Progesterone Receptor; HER2, Human Epidermal Growth Factor Receptor 2; EFS, Event-Free Survival.

Notably, in the HER2+ subgroup, univariate analysis showed that higher SAA levels were associated with poorer EFS (HR = 2.27, 95% CI: 1.00-5.19, p = 0.051), while no significant associations were found for other inflammatory markers. After multivariate adjustment, higher SAA remained independently associated with poorer EFS (HR = 2.53, 95% CI: 1.06–6.07, p = 0.037). In the HER2- subgroup, no significant associations between these inflammatory markers and EFS were observed (**Table 4**).

**Table 4:** Univariate and Multivariate Analyses of EFS Stratified by HER2 Status.

Variable	N	HER2+ subgroup				N	HER2- subgroup			
		Univariate analysis		Multivariate analysis			Univariate analysis		Multivariate analysis	
		HR (95%CI)	p-value	HR (95%CI)	p-value		HR (95%CI)	p-value	HR (95%CI)	p-value
Age, years	155	1.00 (0.96, 1.04)	0.900			183	1.01 (0.97, 1.05)	0.654		
BMI	155	0.87 (0.74, 1.01)	0.073	0.77 (0.64, 0.92)	0.004	183	0.97 (0.86, 1.09)	0.576		
T stage										
≤T2	115	1		1		146	1		1	
≥T3	38	4.17 (1.83, 9.52)	<0.001	1.33 (0.50, 3.57)	0.567	31	2.69 (1.20, 6.04)	0.016	2.09 (0.84, 5.19)	0.111
N stage										
<N2	128	1		1		162	1		1	
≥N2	27	5.12 (2.25, 11.60)	<0.001	0.84 (0.29, 2.39)	0.739	21	4.15 (1.79, 9.63)	<0.001	3.71 (1.58, 8.73)	0.003
M stage										
M0	130	1		1		174	1		1	
M1	25	12.90 (5.47, 30.60)	<0.001	0.86 (0.31, 2.39)	0.774	9	5.04 (1.73, 14.70)	0.003	2.49 (0.73, 8.44)	0.143
Ki67										
<40%	71	1				95	1			
≥40%	81	1.19 (0.52, 2.73)	0.674			87	0.86 (0.39, 1.89)	0.715		
ER status										
Negative	80	1				63	1			
Positive	75	1.21 (0.54, 2.75)	0.643			120	0.56 (0.25, 1.22)	0.142		
PR status										
Negative	95	1				74	1		1	
Positive	60	1.63 (0.72, 3.72)	0.244			109	0.37 (0.16, 0.83)	0.016	0.41 (0.18, 0.94)	0.035
SAA										
≤2.06mg/L	115	1		1		110	1			
>2.06mg/L	40	2.27 (1.00, 5.19)	0.051	2.53 (1.06, 6.07)	0.037	73	1.11 (0.51, 2.42)	0.786		
NLR										
≤2.50	110	1				116	1			
>2.50	45	1.24 (0.53, 2.94)	0.621			67	1.13 (0.51, 2.49)	0.766		
PLR										
≤162.89	101	1				128	1			
>162.89	54	1.66 (0.73, 3.77)	0.223			55	1.68 (0.77, 3.66)	0.193		
SII										
≤650.66	112	1				114	1			
>650.66	43	0.69 (0.26, 1.87)	0.472			69	1.44 (0.67, 3.11)	0.354		

CI, Confidence Interval; HR, Hazard Ratio; SAA, serum amyloid A; NLR, neutrophil/lymphocyte ratio; PLR, platelet/lymphocyte ratio; SII, neutrophil ×platelet/lymphocyte ratio; BMI, Body Mass Index; ER, Estrogen Receptor; PR, Progesterone Receptor; HER2, Human Epidermal Growth Factor Receptor 2; EFS, Event-Free Survival.

### Association between inflammatory markers and OS

OS analysis included all 348 patients. In the overall cohort, all four inflammatory markers were significantly associated with worse OS (SAA: p = 0.030; NLR: p = 0.032; PLR: p = 0.015; SII: p = 0.012) (**Figure 1**). After adjusting for clinical confounders in multivariate analysis, higher SAA (HR = 2.67, 95% CI: 1.14–6.26, p = 0.024) and higher SII (HR = 2.65, 95% CI: 1.11–6.32, p = 0.028) remained independently associated with poorer OS.(**Table 5**).

**Table 5:** Univariate and multivariate analyses of OS.

Variable	N	Univariate analysis		Multivariate analysis	
		HR (95%CI)	p-value	HR (95%CI)	p-value
Age, years	348	1.02 (0.98, 1.06)	0.442		
BMI	348	0.95 (0.83, 1.09)	0.439		
T stage					
≤T2	267	1		1	
≥T3	73	3.81 (1.65, 8.80)	0.002	1.61 (0.58, 4.47)	0.364
N stage					
<N2	296	1		1	
≥N2	52	6.42 (2.78, 14.80)	<0.001	3.61 (1.21, 10.80)	0.021
M stage					
M0	310	1		1	
M1	38	5.23 (2.19, 12.50)	<0.001	1.76 (0.53, 5.84)	0.352
Ki67					
<40%	171	1		1	
≥40%	173	2.49 (0.97, 6.39)	0.057	2.14 (0.81, 5.69)	0.126
ER status					
Negative	150	1			
Positive	198	0.48 (0.20, 1.16)	0.105		
PR status					
Negative	176	1			
Positive	172	0.54 (0.22, 1.33)	0.181		
HER2 status					
Negative	188	1			
Positive	160	0.73 (0.31, 1.72)	0.475		
SAA					
≤2.06mg/L	232	1		1	
>2.06mg/L	116	2.54 (1.10, 5.88)	0.030	2.67 (1.14, 6.26)	0.024
NLR					
≤2.50	232	1			
>2.50	116	2.50 (1.08, 5.79)	0.032		
PLR					
≤162.89	232	1			
>162.89	116	2.87 (1.23, 6.73)	0.015		
SII					
≤650.66	232	1		1	
>650.66	116	2.98 (1.27, 6.98)	0.012	2.65 (1.11, 6.32)	0.028

CI, Confidence Interval; HR, Hazard Ratio; SAA, serum amyloid A; NLR, neutrophil/lymphocyte ratio; PLR, platelet/lymphocyte ratio; SII, neutrophil ×platelet/lymphocyte ratio; BMI, Body Mass Index; ER, Estrogen Receptor; PR, Progesterone Receptor; HER2, Human Epidermal Growth Factor Receptor 2; OS, overall survival. Multivariate model: Adjusted for T, N, M stage, Ki67, SAA and SII.

In the HER2+ subgroup, univariate analysis showed that higher SAA was associated with worse OS (HR = 5.67, 95%CI: 1.42-22.70, p = 0.014). Multivariate analysis further identified higher SAA as the sole independent factor associated with worse OS (HR = 4.68, 95% CI: 1.11–19.70, p = 0.035). In the HER2- subgroup, no statistically significant associations between inflammatory markers and OS were observed (**Table 6**).

**Table 6:** Univariate and Multivariate Analyses of OS Stratified by HER2 Status.

Variable	N	HER2+ subgroup					HER2- subgroup				
		Univariate analysis		Multivariate analysis		N	Univariate analysis		Multivariate analysis	
		HR (95%CI)	p-value	HR (95%CI)	p-value		HR (95%CI)	p-value	HR (95%CI)	p-value
Age, years	160	1.05 (0.99, 1.12)	0.112			188	0.99 (0.94, 1.05)	0.775		
BMI	160	0.93 (0.74, 1.17)	0.537			188	0.96 (0.82, 1.13)	0.639		
T stage										
≤T2	117	1		1		150	1			
≥T3	41	10.80 (2.25, 52.20)	0.003	3.60 (0.57, 22.60)	0.173	32	2.34 (0.72, 7.65)	0.158		
N stage										
<N2	130	1		1		166	1		1	
≥N2	30	6.05 (1.62, 22.60)	0.007	1.46 (0.28, 7.77)	0.654	22	8.95 (2.96, 27.00)	<0.001	14.40 (4.39, 47.20)	<0.001
M stage										
M0	131	1		1		179	1		1	
M1	29	10.20 (2.55, 40.90)	0.001	3.97 (0.64, 24.50)	0.138	9	4.61 (1.01, 20.90)	0.048	5.60 (1.05, 29.80)	0.043
Ki67										
<40%	75	1				96	1			
≥40%	82	3.29 (0.68, 15.80)	0.137			91	1.73 (0.51, 5.83)	0.376		
ER status										
Negative	83	1				67	1		1	
Positive	77	2.19 (0.55, 8.76)	0.268			121	0.13 (0.03, 0.61)	0.009	0.02 (0.00, 2.05)	0.097
PR status										
Negative	98	1				78	1		1	
Positive	62	1.99 (0.53, 7.41)	0.305			110	0.17 (0.04, 0.77)	0.022	4.17 (0.04, 412)	0.543
SAA										
≤2.06mg/L	117	1		1		115	1			
>2.06mg/L	43	5.67 (1.42, 22.70)	0.014	4.68 (1.11, 19.70)	0.035	73	1.37 (0.46, 4.07)	0.574		
NLR										
≤2.50	112	1				120	1			
>2.50	48	2.98 (0.80, 11.10)	0.103			68	2.30 (0.77, 6.86)	0.137		
PLR										
≤162.89	102	1				130	1			
>162.89	58	3.56 (0.89, 14.30)	0.072			58	2.54 (0.85, 7.58)	0.095		
SII										
≤650.66	113	1		1		119	1		1	
>650.66	47	3.04 (0.82, 11.30)	0.098	2.12 (0.54, 8.30)	0.279	69	2.88 (0.94, 8.82)	0.064	3.14 (0.98, 10.10)	0.055

CI, Confidence Interval; HR, Hazard Ratio; SAA, serum amyloid A; NLR, neutrophil/lymphocyte ratio; PLR, platelet/lymphocyte ratio; SII, neutrophil ×platelet/lymphocyte ratio; BMI, Body Mass Index; ER, Estrogen Receptor; PR, Progesterone Receptor; HER2, Human Epidermal Growth Factor Receptor 2; OS, overall survival. Model (HER2+): Adjusted for T, N, M stage, SAA and SII. Model (HER2-): Adjusted for N, M stage, ER, PR and SII.

## Discussion

While traditional prognostic factors like tumor stage and molecular subtype are well-established (20), the role of the inflammatory microenvironment in breast cancer progression and treatment response is gaining significant recognition. Numerous studies have explored the prognostic value of inflammatory markers including NLR, PLR, and SII (6, 8, 15, 21). However, research on SAA in breast cancer remains limited, particularly regarding its association with treatment outcomes. This study provides the first comprehensive evaluation of the relationship between these inflammatory biomarkers and treatment outcomes as well as prognosis in breast cancer.

We observed a significant negative association between SAA levels and ORR. This suggests that elevated SAA may reflect poorer short-term response to chemotherapy. ORR, which reflects immediate tumor shrinkage, may be particularly sensitive to inflammation-mediated changes in the tumor microenvironment (22–24). As a acute-phase protein, elevated SAA may promote chemotherapy resistance by activating pro-survival pathways (e.g., NF-κB/STAT3) and recruiting immunosuppressive cells (e.g., MDSCs, TAMs), thereby shaping an immunosuppressive microenvironment (12, 25). This may explain the lower ORR observed in patients with high SAA levels.

Notably, SAA was independently associated with ORR and OS, but not with pCR and EFS in the overall population. These differences may relate to variations in statistical power, endpoint definitions, and population composition. First, the sample size in the neoadjuvant subset was modest, which could limit statistical power—especially for pCR, a more stringent pathological outcome. Second, the overall EFS analysis included a notable proportion of HR+ patients (57.2%), a subgroup with generally favorable prognosis and lower event rates (e.g., HR+/HER2-: 11.4%; TNBC: 18.1%). This lower-risk profile may dilute the ability to detect a prognostic association of SAA in the overall population, suggesting the lack of association with EFS could reflect cohort composition rather than biological irrelevance. Larger, subtype-stratified studies are needed to clarify these potential context-dependent effects.

In the overall population, while univariate analysis showed associations between NLR, PLR, SII and poorer OS, only SII retained an independent association with OS after adjusting for clinical confounders alongside SAA. This finding differs from prior reports, where PLR has been suggested to better predict pCR and DFS in Luminal B-like (HER2−) patients (7), lower SII has been linked to longer DFS in HER2+ cohorts (21), and elevated preoperative NLR/PLR has been associated with worse OS and DFS (26). Conversely, Wu et al. reported that only SIRI among several inflammatory indices consistently predicted outcomes in HER2+ patients (27). These discrepancies may stem from differences in population characteristics, molecular subtypes, and treatment measures. Several factors may explain the inconsistent prognostic utility: First, variability in cutoff values—while we used upper tertiles for stratification, other studies employed ROC-derived thresholds (e.g., NLR >3 versus our 2.5) or clinical guidelines, resulting in different risk group definitions (26). Second, population heterogeneity may have introduced residual confounding despite multivariable adjustment. This population-level “inflammatory background noise” particularly affects unstable cell count-based indices like NLR and PLR. In contrast, SAA, a protein responsive to specific cytokine pathways, may exhibit greater specificity in reflecting inflammatory activity. Notably, SII—which integrates neutrophils, platelets, and lymphocytes—may offer a more comprehensive assessment of systemic inflammation, potentially explaining its retained association with OS in multivariate analysis. These suggests that within complex inflammatory networks, integrating multi-lineage cellular information may offer more robust insights than single ratios (NLR, PLR).

The persistence of an independent association between SAA and outcomes after adjusting for TNM stage suggests SAA may reflect biological processes beyond conventional staging. This could stem from its dual role as both a systemic acute−phase reactant and a locally active effector within the tumor microenvironment (TME) (25). Through pathways such as TLR4/NF-κB, SAA may sustain a pro-inflammatory cytokine feedback loop (e.g., IL-6, TNF-α) (11, 25, 28), while also promoting an immunosuppressive TME via mechanisms including immune-cell recruitment and PD-L1 upregulation (12, 29). Such systemic-local interplay might help explain the observed links between elevated SAA, lower ORR, and poorer OS. In an exploratory subgroup analysis of HER2+ patients, elevated SAA was associated with poorer outcomes, specifically for worse EFS and OS. This subtype-specific association may involve HER2-driven activation of PI3K/AKT and MAPK pathways (30, 31) synergizing with SAA-mediated TLR4/NF-κB signaling, potentially amplifying inflammatory responses and contributing to therapy resistance (32). Given the retrospective design and limited sample size in this subgroup, these observations warrant further validation in larger prospective HER2+ cohorts.

This study has several limitations. First, as a single-center retrospective study, selection bias may limit generalizability, underscoring the need for validation in prospective multicenter cohorts. Second, the limited sample size compromised statistical power for subgroup analyses (e.g., triple-negative breast cancer, pCR) and prevented chemotherapy regimen-specific comparisons. Third, dynamic changes in SAA and related inflammatory markers (e.g., CRP, IL-6) were not evaluated. Fourth, the internally derived tertile-based cutoffs require external validation.

In summary, this study suggests that SAA may serve as an independent biomarker associated with both prognosis and treatment response in breast cancer patients, particularly in the HER2+ subtype. Prospective multicenter validation, dynamic SAA monitoring for treatment guidance, and mechanistic elucidation warrant future investigation.

## Data Availability

The raw data supporting the conclusions of this article will be made available by the authors, without undue reservation.
