# Supplementary material for: Correlation analysis of serum amyloid A, neutrophil-lymphocyte ratio, platelet-lymphocyte ratio, and systemic immune-inflammation index with neoadjuvant therapy efficacy and prognosis in breast cancer
Source: Front Oncol. 2026 Mar 4;16:1695123. doi: 10.3389/fonc.2026.1695123 (PMC12995658; doi:10.3389/fonc.2026.1695123)
Supplement: Supplementary file 1 [file Table1.docx]

Supplementary Table 1. Association of Inflammatory Markers with pCR Stratified by HER2 Status

Note: pCR, pathological complete response.

Supplementary Table 2. Distribution of neoadjuvant treatment regimens in 113 breast cancer patients stratified by SAA level and HER2 status

**Notes:** SAA, Serum amyloid A; Dual-targeted chemotherapy, Anti-HER2 targeted therapy combined with chemotherapy; Single-targeted chemotherapy, Single anti-HER2 targeted therapy combined with chemotherapy; AI, Aromatase inhibitor; OFS, Ovarian function suppression; SERM, Selective estrogen receptor modulator; (See the end of the text for full abbreviation terms.)

Supplementary Table 3. Logistic regression analysis of ORR in neoadjuvant-treated breast cancer patients

**Notes:** CI, Confidence Interval; OR, Odds Ratio.

SAA, serum amyloid A; NLR, neutrophil/ lymphocyte ratio; PLR, platelet/lymphocyte ratio; SII, neutrophil ×platelet/lymphocyte ratio; BMI, Body Mass Index; TNM, Tumor-Node-Metastasis staging system; ER, Estrogen Receptor; PR, Progesterone Receptor; CT/ET, Chemotherapy/Endocrine Therapy; DTC, Dual-Targeted Chemotherapy; STC, Single-Targeted Chemotherapy; ORR (objective response rate), Complete response (CR) + Partial response (PR).

Supplementary Table 4. AIC and C‑Index Comparison in OS Multivariate Cox Analysis (Overall Population)

| Model | Variable | AIC | C_index | Delta_AIC | Rank |
| --- | --- | --- | --- | --- | --- |
| Model_1 | SAA+SII | 224.5869 | 0.81251 | 0 | 1 |
| Model_2 | SAA+NLR+SII | 226.3273 | 0.8199203 | 1.74 | 2 |
| Model_3 | SAA+PLR+SII | 226.3528 | 0.8173705 | 1.77 | 3 |
| Model_4 | SAA+PLR | 226.8942 | 0.8027888 | 2.31 | 4 |
| Model_5 | SAA+NLR | 227.7854 | 0.7888446 | 3.2 | 5 |
| Model_6 | ALL | 228.069 | 0.8211952 | 3.48 | 6 |
| Model_7 | SAA+NLR+PLR | 228.4737 | 0.8108367 | 3.89 | 7 |

Supplementary Table 5. Results of Different Multivariate Cox Models for Overall Survival (Overall Population)

**Note:** For Table 5 in the main manuscript (revised version)—which presents the multivariate analysis of overall survival (OS) stratified by HER2 status—the findings were consistent with the aforementioned OS analysis of the overall population. Based on the Akaike Information Criterion (AIC) and discriminative ability (C‑index) (Supplementary Tables 6 and 7), it was ultimately decided to retain only the SII variable in the final model (see the revised Table 6 in the manuscript).

Supplementary Table 6. AIC and C‑Index Comparison in OS Multivariate Cox Analysis (HER2+ Population)

| Model | Variable | AIC | C_index | Delta_AIC | Rank |
| --- | --- | --- | --- | --- | --- |
| Model_1 | SAA+SII | 78.47679 | 0.871814 | 0 | 1 |
| Model_2 | SAA+PLR | 78.9302 | 0.8689 | 0.45 | 2 |
| Model_3 | SAA+PLR+SII | 80.4229 | 0.871814 | 1.95 | 3 |

Supplementary Table 7. AIC and C‑Index Comparison in OS Multivariate Cox Analysis (HER2- Population)

| Model | Variable | AIC | C_index | Delta_AIC | Rank |
| --- | --- | --- | --- | --- | --- |
| Model_1 | SII | 112.1715 | 0.803058 | 0 | 1 |
| Model_2 | PLR | 112.7161 | 0.783873 | 0.54 | 2 |
| Model_3 | PLR+SII | 114.0889 | 0.806355 | 1.92 | 3 |

Supplementary Figure 1. Correlation, collinearity, and model fit comparison of inflammatory indices

**Note:** Supplementary Figure 1. Correlation, collinearity, and model fit comparison of inflammatory indices. a-c: Pairwise Spearman correlation of continuous NLR, PLR, and SII variables; d: Variance inflation factor (VIF) for continuous variables in multivariate model; e: Akaike Information Criterion (AIC) comparison across model combinations; f: C-index comparison across models (All models included SAA). ALL indicates the model including all four indices (SAA+NLR+PLR+SII).

Supplementary Figure 2. ROC curves of SAA, NLR, PLR, and SII for predicting Treatment Response and Survival Outcomes in Breast Cancer

**Note:** A. ROC curves of SAA, NLR, PLR, and SII for predicting ORR; B. ROC curves of SAA, NLR, PLR, and SII for predicting pCR; C. ROC curves of SAA, NLR, PLR, and SII for predicting EFS; D. ROC curves of SAA, NLR, PLR, and SII for predicting OS. SAA, serum amyloid A; NLR, neutrophil/ lymphocyte ratio; PLR, platelet/lymphocyte ratio; SII, neutrophil ×platelet/lymphocyte ratio; ROC curves: Receiver Operating Characteristic Curve; AUC: Area Under the Curve; ORR: Objective Response Rate; pCR: Pathological Complete Response; EFS: Event-Free Survival; OS: Overall Survival.

**Supplementary Table 2 Abbreviations:**

SAA, serum amyloid A;

DTC, dual-target therapy combined with chemotherapy;

TCbHP, docetaxel, carboplatin, trastuzumab, pertuzumab;

THP, docetaxel, trastuzumab, pertuzumab;

AC_THP, doxorubicin/cyclophosphamide followed by docetaxel/trastuzumab/pertuzumab;

ECPyr_THPyr, epirubicin/cyclophosphamide/paclitaxel followed by trastuzumab/pertuzumab;

STC, single-target therapy combined with chemotherapy;

EC_TH, epirubicin/cyclophosphamide followed by docetaxel/trastuzumab;

TCbH, docetaxel, carboplatin, trastuzumab;

CT, chemotherapy;

TAC, docetaxel, doxorubicin, cyclophosphamide;

AC/TC/TX, doxorubicin/cyclophosphamide, docetaxel/cyclophosphamide, or docetaxel/capecitabine (non-standardized regimens);

ET, endocrine therapy; AI, aromatase inhibitor;

OFS_AI, ovarian function suppression plus aromatase inhibitor;

OFS_SERM, ovarian function suppression plus selective estrogen receptor modulator.
